# Quantum simulation of noise-slowed domain-wall motion in a phenomenological spin model

**DOI:** 10.1038/s41598-026-63377-3

**Published:** 2026-07-23

**Authors:** Jaka Vodeb

**Affiliations:** 1 Department of Complex Matter, Jožef Stefan Institute, Jamova 39, 1000 Ljubljana, Slovenia; 2 CENN Nanocenter, Jamova 39, 1000 Ljubljana, Slovenia

**Keywords:** Materials science, Physics

## Abstract

Metastable domain-wall networks in correlated materials can relax on timescales vastly longer than the microscopic processes that move their elementary constituents. Motivated by the slow domain-wall rearrangements observed in 1T-TaS$_2$, we study a phenomenological spin model designed to isolate the interplay between local domain-wall hopping and environmental noise. The model contains discrete registry-like configurations, energetic costs for domain walls, and a transverse-field-induced hopping channel, and is implemented on a superconducting quantum annealer with more than 2000 logical degrees of freedom. A Schrieffer–Wolff analysis shows that the intrinsic hopping amplitude arises at second order in the transverse field and is therefore fast on device timescales. However, the measured reconfiguration rate saturates to a finite value at low effective temperature and depends only weakly on the hopping amplitude. This indicates that domain-wall motion is not limited by bare tunnelling, but by quasi-static longitudinal-field noise that detunes neighboring configurations and intermittently permits local hopping events. These results demonstrate how noise can slow domain-wall motion in a minimal quantum-simulation model and suggest a possible mechanism for ultra-slow relaxation in charge-ordered materials.

## Introduction

Quantum simulation has become a powerful tool for probing many-body dynamics that are difficult to access by either direct experiment or classical computation. Recent progress across diverse platforms has enabled controlled studies of quantum chemistry^1,2^, quantum criticality^3,4^, lattice gauge theories^5,6^, and disorder-driven localization^7^. An important emerging use of programmable quantum devices is not only to reproduce idealized Hamiltonians, but also to isolate dynamical mechanisms that may be relevant to complex quantum materials.

A central challenge in correlated materials is the emergence of relaxation timescales that are vastly longer than the underlying microscopic electronic processes. The layered dichalcogenide 1T-TaS$_2$ provides a prominent example. At low temperatures, it forms a commensurate charge-density wave (CDW) composed of localized polarons arranged in a triangular superlattice^8–11^. Scanning tunnelling microscopy (STM) reveals extended networks of narrow domain walls separating distinct CDW domains^12^. Time-resolved STM measurements show that relaxation of these metastable textures proceeds via discrete rearrangements of domain walls over seconds to hours, despite electronic energy scales corresponding to femtosecond timescales^12^. At sufficiently low temperatures, the relaxation becomes weakly temperature dependent, suggesting that thermal activation alone does not determine the reconfiguration rate^12^.

This phenomenology raises a general mechanistic question: how can intrinsically fast local rearrangement processes generate ultra-slow domain-wall motion? In particular, if domain-wall motion proceeds through local hopping events, the observed timescale may be controlled not by the bare hopping amplitude itself, but by environmental fluctuations that modulate the local energy mismatch between neighboring configurations. This possibility is difficult to isolate directly in the material, where electron–phonon coupling, long-range Coulomb interactions, disorder, and lattice degrees of freedom are all intertwined and their tunability limited.

Here we address this question using a phenomenological spin-model quantum simulation. Motivated by the domain-wall phenomenology of 1T-TaS$_2$, we construct a minimal transverse-field Ising model on a triangular lattice that contains the ingredients needed for discrete domain-wall rearrangements: registry-like classical configurations, energetic costs for domain walls, and a transverse-field-induced hopping channel. The model is not intended as a microscopic derivation of the electronic Hamiltonian of 1T-TaS$_2$. Rather, it provides a controlled setting in which the interplay between local domain-wall hopping and noise can be studied directly.

Using a large-scale implementation on a superconducting quantum annealer, we show that the intrinsic hopping processes generated by the transverse field are fast, but that domain-wall motion is strongly slowed by quasi-static longitudinal-field noise. A Schrieffer–Wolff analysis shows that the relevant local hopping amplitude arises at second order in the transverse field. However, the measured reconfiguration rate saturates to a finite value at low effective temperature and depends only weakly on the hopping amplitude. This indicates that the relaxation is not limited by bare tunnelling, but by noise-induced detuning that suppresses hopping between neighboring domain-wall configurations.

Our results establish a mechanism by which noise can slow domain-wall motion in a phenomenological quantum-simulation model. For 1T-TaS$_2$, this provides a concrete candidate explanation for the extreme separation between microscopic electronic timescales and the observed slow relaxation of domain-wall networks. More broadly, the work illustrates how noisy quantum simulators can be used to test mechanistic hypotheses about non-equilibrium relaxation in correlated quantum materials.

## Results

### Domain-wall phenomenology and phenomenological spin-model construction

**Fig. 1:**
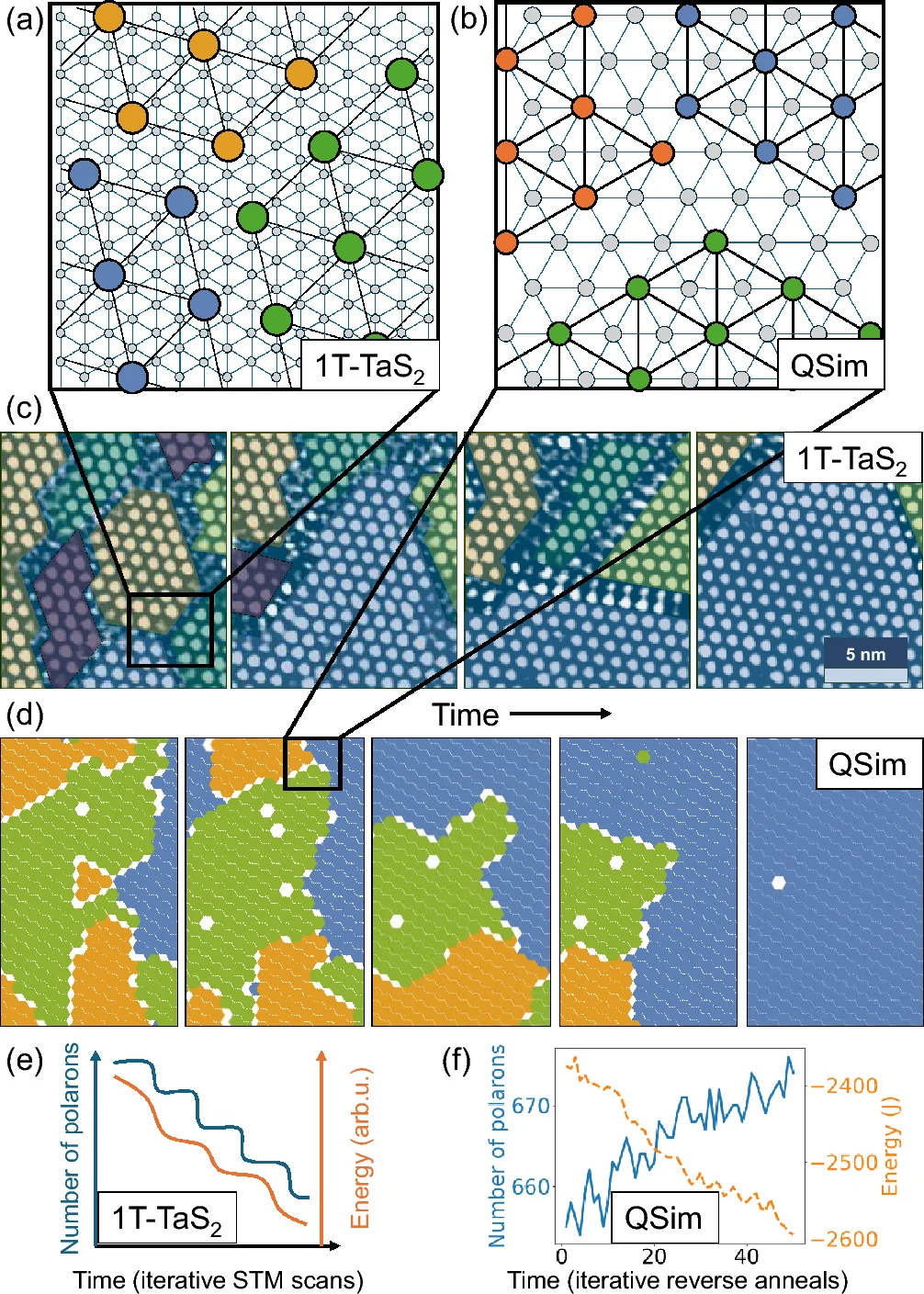
Domain structures and relaxation in 1T-TaS$_2$ and in quantum simulation. (**a**) Representative CDW domain configuration in 1T-TaS$_2$, where colors denote the thirteen distinct commensurate CDW domains related by relative translations of the 1/13-filled polaronic superlattice. Extended domain walls separate regions of different CDW domains and store excess interaction energy. (**b**) Corresponding domain configuration obtained in the quantum simulation on a quantum annealer. Colors label the three degenerate domains of the effective triangular-lattice transverse-field Ising model. Despite the reduced number of domain types, the local domain-wall geometry is the same as in the material. Black outlines and lines show (a) and (b) as insets of (c) and (d). (**c**) Time-resolved STM images of 1T-TaS$_2$ showing slow relaxation of an initially non-equilibrium domain-wall network toward the ordered ground state. (**d**) Representative sequence of configurations from the quantum simulation obtained via iterative annealing cycles, showing domain-wall annihilation and coarsening. (**e**) Schematic evolution of polaron density and interaction energy of STM measurements during relaxation in 1T-TaS$_2$, where domains are typically offset closer together due to an excess of charge and gradually reconfigure into a single domain through charge leakage. (**f**) Evolution of polaron density and interaction energy obtained from the quantum simulation. The system initializes with missing charge, evidenced by a typical neighboring domain offset moving them away from each other, and leading to an increase in polaron number during relaxation. Despite this global difference, the local polaronic dynamics within domain walls are similar between experiment and simulation.

At low temperatures, 1T-TaS$_2$ forms a commensurate charge-density wave (CDW) that can be viewed as a 1/13-filled triangular Wigner crystal of localized electrons, or polarons, corresponding to one electron per thirteen Ta atoms^8–11,13^. The uniform CDW configuration is the ordered ground state, while STM experiments reveal extended networks of narrow domain walls separating regions with different CDW registries^12^, shown in Fig. 1a. Time-resolved STM measurements show that relaxation of these metastable textures proceeds via discrete rearrangements of domain walls over seconds to hours, despite microscopic electronic timescales being on the order of femtoseconds^12^. This phenomenology motivates a reduced description focused on the motion of domain-wall segments rather than on the full electronic structure of the material (Fig. 1b).

In 1T-TaS$_2$, the commensurate CDW supports thirteen symmetry-related domain registries associated with translations of the 1/13 polaron superlattice. Across a domain wall, the local CDW registry changes by one of a small set of displacement vectors, with nearest-neighbor registry shifts dominating the observed domain-wall geometry and its evolution, shown in Fig. 1c. The key ingredients for modeling the observed relaxation are therefore discrete registry-like configurations, an energetic cost associated with domain walls, and a mechanism that allows domain-wall segments to hop between nearby configurations.

We encode these ingredients in a phenomenological transverse-field Ising model on a triangular lattice,

1 $$\begin{aligned} \hat{H}_{\textrm{TFIM}} = J\sum _{\langle i,j\rangle }\hat{\sigma }_i^z\hat{\sigma }_j^z - h_x\sum _i\hat{\sigma }_i^x + h_z\sum _i\hat{\sigma }_i^z . \end{aligned}$$

Here, the Ising variables are not electronic spins. They represent a reduced binary degree of freedom from which domain registries and domain walls are constructed. The antiferromagnetic interaction *J>0* penalizes locally unfavorable registry patterns and assigns an energy cost to domain-wall configurations. The longitudinal field $h_z$ acts as a chemical-potential term that selects the effective 1/3 ordered manifold. The transverse field $h_x$ introduces a hopping channel between nearby classical configurations, allowing domain-wall segments to rearrange (Fig. 1d).

The model reduces the degeneracy structure of the material. Whereas 1T-TaS$_2$ has thirteen symmetry-related CDW registries at 1/13 filling, the triangular-lattice spin model has three symmetry-related ordered states at effective 1/3 filling. This reduction means that Eq. (1) should not be interpreted as a microscopic Hamiltonian for 1T-TaS$_2$. Its purpose is instead to isolate the local kinematics and energetics of nearest-neighbor domain-wall rearrangements. The experimentally relevant registry shifts are represented as boundaries between the symmetry-related ordered states of the spin model, allowing the model to capture the type of local domain-wall motion relevant for relaxation while avoiding the full microscopic complexity of the material.

A second distinction concerns global charge evolution. In STM experiments, relaxation is typically initiated from an electron-doped metastable state, and the total polaron density decreases over time. In the quantum simulation, the initial states are hole-doped relative to the effective ordered configuration, leading instead to an increase in effective polaron density during relaxation (Fig. 1e,f). Despite this difference, both systems relax by reducing interaction and chemical-potential energy stored in the domain-wall network. The comparison is therefore made at the level of domain-wall rearrangement dynamics, not at the level of global charge transport or absolute material timescales.

This phenomenological construction has a practical advantage: it can be implemented directly on a superconducting quantum annealer. The device provides a large-scale open-system realization of Eq. (1), in which the transverse field controls the intrinsic hopping amplitude and device noise produces slowly varying longitudinal-field detuning. This makes it possible to study how an otherwise fast local hopping process becomes slowed by noise. In the following sections, we analyze the effective hopping generated by the transverse field and then use the quantum simulation to show that the observed domain-wall motion is controlled primarily by noise-induced detuning rather than by the bare hopping amplitude.

### Effective dynamics within the domain-wall manifold

We next analyze how the transverse field in Eq. (1) generates motion of domain-wall segments in the weak-transverse-field regime. We consider $J\gg h_x$, where the classical Ising terms define well-formed ordered regions and energetically costly domain walls. In this limit, the transverse field acts as a perturbation that connects nearby classical configurations.

We decompose Eq. (1) as

$$H_{\textrm{TFIM}}=H_0+V,$$

with

$$H_0 = J\sum _{\langle i,j\rangle }\hat{\sigma }_i^z\hat{\sigma }_j^z + h_z\sum _i\hat{\sigma }_i^z, \qquad V = -h_x\sum _i\hat{\sigma }_i^x .$$

The unperturbed Hamiltonian $H_0$ assigns an energy to each classical spin configuration according to its domain-wall geometry and local occupation imbalance. The perturbation *V* flips individual spins and therefore either creates or removes local defects, or acts virtually to connect two configurations within the same domain-wall manifold.

**Fig. 2:**
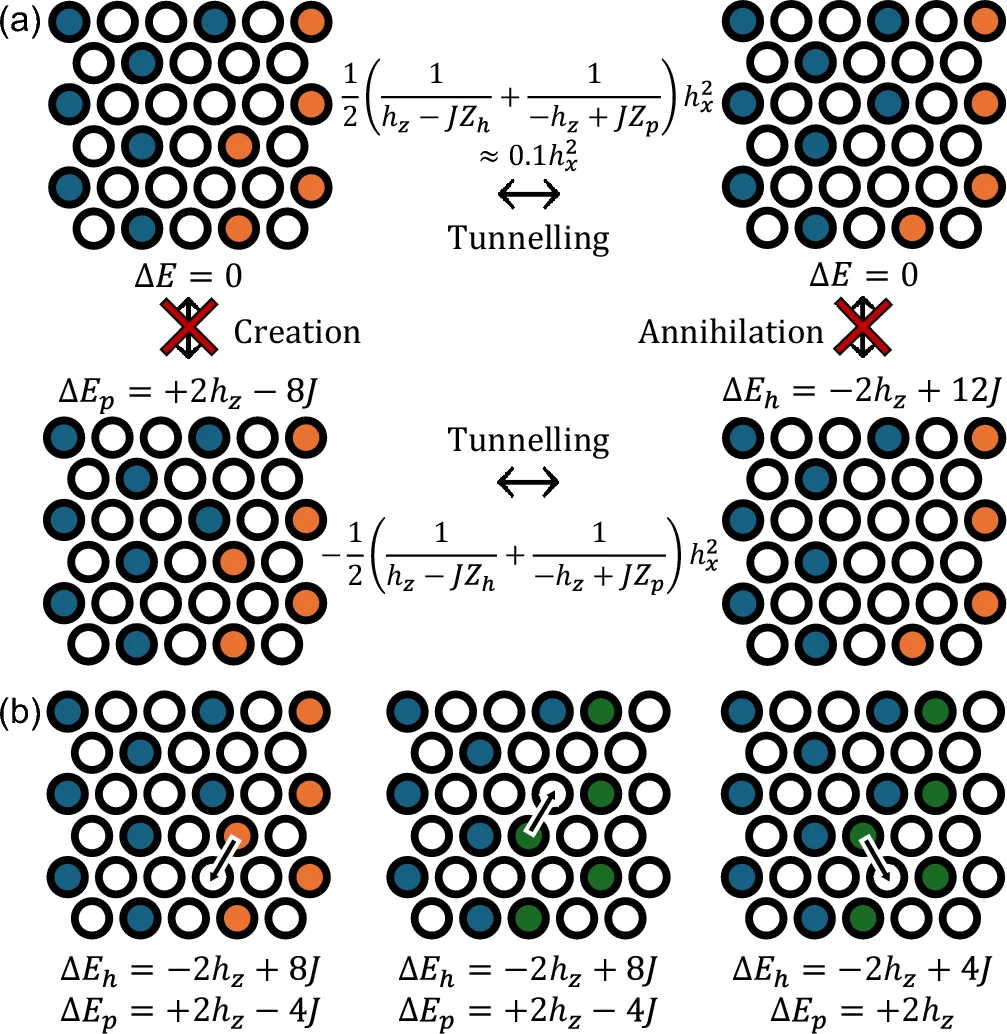
Effective local hopping processes within domain walls. (**a**) Representative local configurations involved in domain-wall motion in the transverse-field Ising model. Within the domain-wall manifold, the leading allowed rearrangements arise from second-order virtual processes generated by the transverse field. These processes connect locally equivalent configurations through nearest-neighbor hopping events with matrix elements proportional to $h_x^2$. The corresponding virtual excitation energies *Δ E* and effective hopping amplitudes are indicated next to each process. First-order creation or annihilation processes are energetically suppressed, as marked by red crosses. (**b**) Additional domain-wall geometries observed in the quantum simulation. Black arrows indicate the most likely local hopping events within the domain wall. The intermediate virtual states involve either particle annihilation, with energy cost $\Delta E_h$, or particle creation, with energy cost $\Delta E_p$. The variation of these energy costs reflects the dependence of the effective hopping amplitude on the local spin environment.

Our aim is not to obtain the full low-energy spectrum of the triangular-lattice TFIM. Instead, we focus on the local two-site processes that move a segment of an already existing domain wall. Representative local environments are shown in Fig. 2. In these configurations, two domain-wall states are classically degenerate or nearly degenerate, while the intermediate states reached by a single spin flip correspond to local defect creation or annihilation. Their excitation energies are set by the surrounding spin environment and provide the denominators that enter the effective hopping amplitude.

We integrate out these high-energy intermediate states using a Schrieffer–Wolff transformation^14^. To second order in $h_x$, this yields an effective Hamiltonian acting within the constrained domain-wall manifold,

2 $$\begin{aligned} \hat{H}_{\textrm{eff}} = - \sum _{\langle i,j\rangle } t_{ij} \left( \hat{\sigma }_i^+\hat{\sigma }_j^-+\mathrm {h.c.} \right) + \mathcal {O}(h_x^3), \end{aligned}$$

where

3 $$\begin{aligned} t_{ij}=h_x^2\lambda _{ij}. \end{aligned}$$

The coefficient $\lambda _{ij}$ is controlled by the virtual excitation energies associated with the two possible intermediate states. For the local two-site process derived explicitly in the Methods section,

4 $$\begin{aligned} \lambda _{ij} = \frac{1}{2} \left[ \frac{1}{h_z-JZ_h} + \frac{1}{-h_z+JZ_p} \right] , \end{aligned}$$

where $Z_h$ and $Z_p$ count the neighboring hole-like $(\hat{\sigma }^z=-1)$ and particle-like $(\hat{\sigma }^z=+1)$ spins surrounding the two sites involved in the hopping event. The different values of $Z_h$ and $Z_p$ correspond to the different local domain-wall geometries illustrated in Fig. 2. Thus $\lambda _{ij}$ has units of inverse energy, and variations in the local domain-wall environment lead to spatial variations of the hopping amplitude.

For typical local environments in which the virtual excitation denominators are of order *J*, one obtains $\lambda _{ij}\sim J^{-1}$, $t_{ij}\sim h_x^2/J$. This makes explicit that the effective hopping amplitude is second order in the transverse field and remains an energy scale. First-order processes in $h_x$ correspond to single-spin flips that create or annihilate local defects in the ordered background. These processes are energetically suppressed in the domain-wall configurations considered here, as shown schematically by the forbidden creation and annihilation processes in Fig. 2. The leading processes that move domain-wall segments are therefore the second-order nearest-neighbor hopping events in Eq. (2). In addition, energy relaxation does permit single spin flips, which is a first-order process in $h_x$. However, as shown below, the measured reconfiguration rate depends only weakly on $h_x$, consistent with a noise-controlled mechanism rather than a hopping- or relaxation-limited one.

Whether such a hopping event occurs dynamically depends not only on the intrinsic hopping amplitude $t_{ij}$, but also on the local detuning between the two configurations connected by the hop. We denote this energy mismatch by $\Delta _{ij}$. It receives contributions from slowly varying device-induced longitudinal-field fluctuations^15^. At the level of a local two-state description, the relevant Hamiltonian can be written as

$$H_{ij} = \frac{\Delta _{ij}}{2}\hat{\sigma }^z + t_{ij}\hat{\sigma }^x .$$

When $|\Delta _{ij}|\lesssim t_{ij}$, the two configurations are effectively resonant and hopping proceeds on the intrinsic timescale $\sim 1/t_{ij}\sim J/h_x^2$, which is short compared with the timescale over which $\Delta _{ij}$ fluctuates. This local hopping process is the spin-model analogue of local electronic or polaronic hopping in the material. When $|\Delta _{ij}|\gg t_{ij}$, however, the transition probability is strongly suppressed. Thus, even when the intrinsic hopping amplitude is large enough to produce fast local motion, domain-wall rearrangements can be strongly slowed by detuning.

This suppression can be written explicitly using the two-level dynamics of the local hopping channel. During a window of duration $t_a$ (corresponding to one reverse-annealing cycle in the next section), the probability for a nearest-neighbor domain-wall hopping event is

$$P_{ij}(t_a) = \frac{4t_{ij}^2}{\Delta _{ij}^2+4t_{ij}^2} \sin ^2\left[ \frac{t_a}{2\hbar } \sqrt{\Delta _{ij}^2+4t_{ij}^2} \right] .$$

The prefactor shows that, for $|\Delta _{ij}|\gg t_{ij}$, the maximum transition probability is suppressed as

$$P_{ij}^{\textrm{max}} \sim \frac{4t_{ij}^2}{\Delta _{ij}^2}.$$

The effective probability for a local rearrangement during one activation cycle is therefore controlled by the distribution of detunings sampled by the quasi-static noise landscape,

$$p_{ij} = \left\langle P_{ij}(t_a;\Delta _{ij}) \right\rangle _{\Delta _{ij}},$$

where the average is over the slowly varying values of $\Delta _{ij}$ encountered from cycle to cycle.

This analysis establishes the mechanism studied in the remainder of the paper. The transverse field generates intrinsic local hopping of domain-wall segments with amplitude $t_{ij}\sim h_x^2/J$. Quasi-static longitudinal-field noise modulates the energy mismatch $\Delta _{ij}$ between the two lattice sites involved in a nearest-neighbor hopping event and thereby controls the probability $p_{ij}$ that the hop occurs during a given activation window. The observed relaxation rate is consequently expected to depend not only on the bare hopping amplitude, but also on the statistics of the noisy detuning landscape.

### Quantum simulation protocol and Hamiltonian parametrization

To study noise-slowed domain-wall motion, we implement the phenomenological spin model of Eq. (1) on a programmable superconducting quantum annealer (D-Wave Advantage6.1). The device realizes an open quantum system governed by the time-dependent two-field quantum Ising Hamiltonian

5 $$\begin{aligned} \hat{H}_{\textrm{DW}}(s) = -\frac{A(s)}{2}\sum _i \hat{\sigma }_i^x + \frac{B(s)}{2} \left( \sum _{i,j} J_{ij}\hat{\sigma }_i^z\hat{\sigma }_j^z + \sum _i h_i^z \hat{\sigma }_i^z \right) , \end{aligned}$$

where *s∈ [0,1]* is the annealing schedule parameter. The functions *A*(*s*) and *B*(*s*) set the transverse-field and longitudinal Ising energy scales, respectively. The Pauli operators act on logical spins encoding the reduced registry variables introduced above.

The annealer is not an isolated quantum system: it operates in the presence of environmental coupling, finite temperature, and low-frequency device noise. This open-system character is central to the present work, because it allows us to study how hopping generated by the transverse field is modified by slowly varying longitudinal-field detuning.

To connect Eq. (5) to Eq. (1), we write $J_{ij}=J\tilde{J}_{ij}$ and $h_i^z=J\tilde{h}_i^z$, where $\tilde{J}_{ij}$ and $\tilde{h}_i^z$ define the dimensionless problem instance and *J* is the dimensionless programmable coupling scale. We set *J=α r*, where *r* controls the ratio between the interaction scale and the transverse-field amplitude. Factoring out *rA*(*s*)/2 gives

6 $$\begin{aligned} \begin{aligned} \frac{\hat{H}_{\textrm{DW}}}{k_\textrm{B}T}&= \frac{A(s)r}{2k_\textrm{B}T} \bigg [ -\frac{1}{r}\sum _i \hat{\sigma }_i^x \\&\quad + \frac{B(s)}{A(s)}\,\alpha \left( \sum _{i,j} \tilde{J}_{ij}\hat{\sigma }_i^z\hat{\sigma }_j^z + \sum _i \tilde{h}_i^z \hat{\sigma }_i^z \right) \bigg ]. \end{aligned} \end{aligned}$$

Choosing *α =A(s)/B(s)* cancels the explicit *B*(*s*)/*A*(*s*) ratio in the dimensionless Hamiltonian. In this parametrization, the effective transverse field is

$$h_x=\frac{1}{r},$$

and the effective temperature parameter is

$$T_\textrm{eff}[s(t)] = \frac{2k_\textrm{B}T}{A(s)r}.$$

Since both $k_\textrm{B}T$ and *A*(*s*) are energies, $T_\textrm{eff}$ is dimensionless. We use it as a convenient characterization of the relative strength of thermal and noise-induced fluctuations compared with the Hamiltonian energy scale, not as an equilibrium temperature of a closed system.

The reduced couplings are chosen as $\tilde{J}_{ij}=1$ for nearest neighbors on the triangular lattice and zero otherwise, implementing antiferromagnetic interactions that penalize domain walls. The reduced longitudinal fields $\tilde{h}_i^z$ encode a chemical-potential term and depend on the local coordination, taking the value 4 for bulk sites with six neighbors and decreasing to 3, 2, 1, or 0 at boundaries, depending on the number of neighbors.

**Fig. 3:**
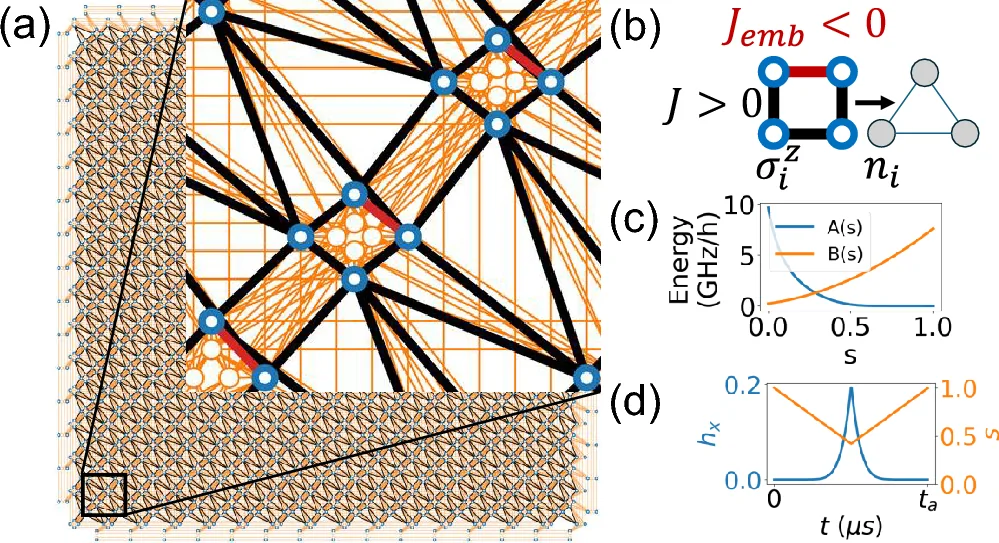
Quantum simulation of domain-wall dynamics on a superconducting quantum annealer. (**a**) Embedding of a triangular lattice with $N_\textrm{L}=2008$ logical sites into the quantum annealer using $N_\textrm{P}=2673$ physical qubits. Each square plaquette of four physical qubits represents a building block triangle of the logical lattice, resulting in a physical-to-logical qubit ratio of 4/3. Inset: detailed view of the embedding. (**b**) Schematic of the embedding couplings. Black links denote antiferromagnetic Ising couplings (*J>0*) that map onto the edges of the logical triangular lattice. Red links indicate strong ferromagnetic embedding couplings ($J_\textrm{emb}<0$) that bind pairs of physical qubits into a single logical qubit by enforcing identical spin states. (**c**) Energy scales of the quantum annealer Hamiltonian as functions of the annealing schedule parameter *s*. The transverse-field term *A*(*s*) and the Ising term *B*(*s*) are shown in units of GHz/*h*, where *h* is Planck’s constant. (**d**) Reverse annealing protocol used to generate relaxation dynamics. Starting from a classical configuration at *s=1* ($h_x=0$), the transverse field is ramped to a target value corresponding to the desired $h_x=1/r$ over half of the total annealing time $t_a=5~\mu s$ and then ramped back to *s=1*. The final configuration of each reverse anneal is used as the initial configuration for the next cycle, producing a discrete-time dynamical evolution analogous to time-resolved STM measurements.

The logical triangular lattice consists of $N_\textrm{L}=2008$ sites and is embedded into the hardware graph using standard minor-embedding techniques, requiring $N_\textrm{P}=2673$ physical qubits. Each elementary triangle of the logical lattice is represented by four physical qubits, with antiferromagnetic couplers implementing logical interactions and strong ferromagnetic embedding couplings binding physical qubits into a single logical spin as shown in Fig. 3a,b.

To generate non-equilibrium relaxation dynamics, we use a reverse annealing protocol. Each cycle begins from a classical configuration at *s=1* (vanishing transverse field). The schedule is then reversed to a target value of *s* over half of the total annealing time $t_a=5~\mu$s, temporarily activating the transverse field and enabling domain-wall rearrangements. The system is subsequently returned to *s=1* for readout. The energy scales *A*(*s*) and *B*(*s*) and the annealing schedule are shown in Fig. 3c,d.

Because the quantum annealer operates as an open system, each reverse-annealing cycle exposes the system to a controlled interval of hopping dynamics in the presence of quasi-static noise in the form of low-frequency fluctuations of small local longitudinal fields. During the reverse ramp, the transverse field is temporarily activated for a duration $t_a$, enabling local domain-wall rearrangements, after which the system is projected back onto a classical configuration through measurement in the $\hat{\sigma }^z$ basis. The final configuration of one cycle serves as the initial state of the next, producing a stroboscopic sequence of configurations connected by short intervals of noisy quantum evolution. This protocol is analogous to time-resolved STM in the limited sense that both approaches generate discrete snapshots of a domain-wall network separated by intervals during which rearrangements may occur. The iteration index therefore plays the role of an effective discrete time variable, allowing us to probe how intrinsic hopping, controlled by $h_x$, competes with noise-induced detuning in determining the probability of domain-wall rearrangement per activation cycle.

### Reconfiguration rates and noise-slowed domain-wall motion

We quantify domain-wall reconfiguration dynamics by the average number of spins that flip between successive reverse-annealing iterations,

7 $$\begin{aligned} R = \frac{1}{N_{\textrm{steps}}} \sum _{t=1}^{N_{\textrm{steps}}} \frac{1}{2} \sum _{i=1}^{N_L} \left| \sigma _i^z(t+1)-\sigma _i^z(t) \right| . \end{aligned}$$

Equivalently, $R/N_L$ gives the fraction of sites whose local registry variable changes per iteration. Each reverse-annealing cycle produces a classical configuration of the domain-wall network, so *R* measures the activity of domain-wall rearrangements between two successive simulated snapshots. Since most spin flips occur at or near domain-wall segments, this quantity probes the reconfiguration dynamics of the network.

This definition is directly analogous to the experimentally extracted polaron reconfiguration rate in 1T-TaS$_2$^12^. In time-resolved STM, successive images of the CDW texture are compared and the number of polarons that change position or registry between two snapshots is used to quantify reconfiguration activity. In the quantum simulation, the iteration index plays the role of a discrete time variable, and *R* measures the number of local registry changes occurring during one reverse-annealing activation cycle. The absolute timescales are not mapped between experiment and simulation; the comparison is made at the level of discrete domain-wall rearrangement activity.

**Fig. 4:**
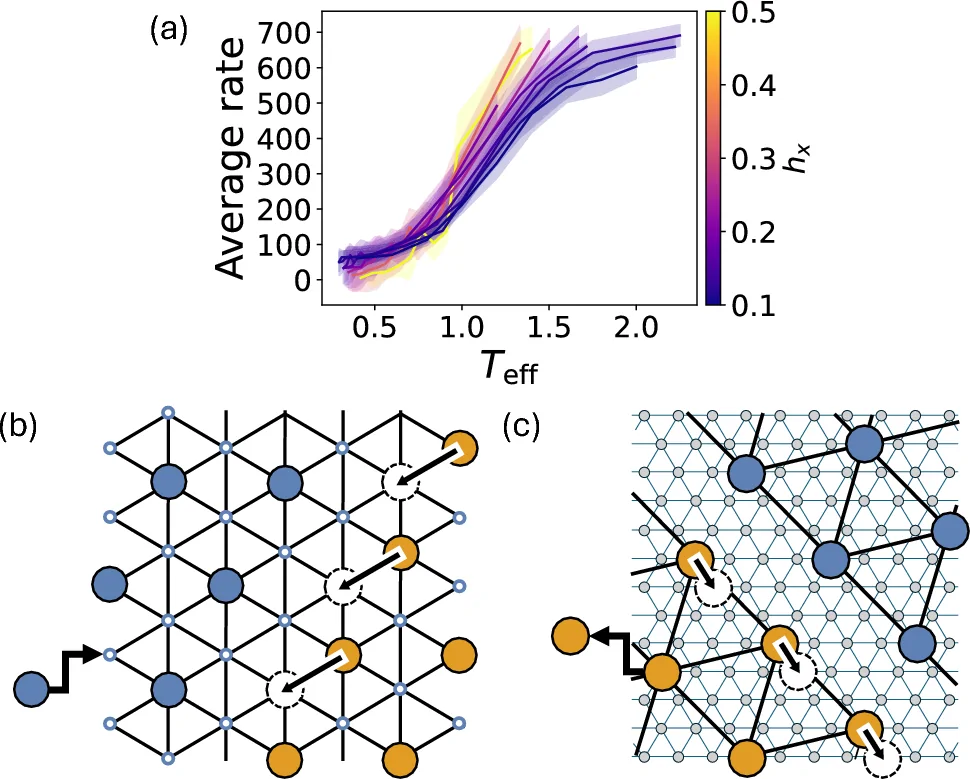
Reconfiguration rates and local mechanisms of noise-slowed domain-wall motion. (**a**) Average reconfiguration rate as a function of the effective temperature parameter $T_\textrm{eff}$ for several transverse-field amplitudes $h_x$. The rate increases strongly at high $T_\textrm{eff}$, consistent with thermally assisted rearrangements, but remains finite at low $T_\textrm{eff}$. The comparatively weak dependence on $h_x$ indicates that the bare hopping amplitude is not the only rate-limiting scale. (**b**) Schematic of elementary rearrangement processes in the phenomenological spin model. Local nearest-neighbor hopping shifts a domain-wall segment by one lattice spacing, while single-spin flips inject particle-like spins and contribute to longer-time relaxation of the domain-wall network. (**c**) Corresponding material-level picture for 1T-TaS$_2$. Local polaronic hopping shifts CDW domain walls, while particle leakage from the domain-wall network changes the local polaron density and contributes to relaxation of the metastable texture. Together, the schematics illustrate how extended domain-wall rearrangements can arise from sequences of local hopping and particle-number relaxation events.

Figure 4a shows *R* as a function of the effective temperature parameter $T_\textrm{eff}$ for several transverse-field amplitudes $h_x$. At high $T_\textrm{eff}$, the reconfiguration rate increases strongly, consistent with thermally assisted rearrangements. At low $T_\textrm{eff}$, however, the rate remains finite instead of vanishing. This finite low-temperature activity indicates that domain-wall motion persists when thermal activation is strongly suppressed, consistent with the presence of quantum hopping processes.

The dependence on $h_x$ is comparatively weak. From the effective-domain-wall analysis above, the transverse field supplies a local hopping channel with amplitude $t_{ij}\sim h_x^2/J$. If the observed reconfiguration rate were limited directly by this bare hopping matrix element, or even by single-spin-flip relaxation ($\sim h_x$), one would expect a much stronger dependence on $h_x$. Instead, Fig. 4a shows that by changing $h_x$ we do not resolve a systematic dependence of *R* on $h_x$ within the uncertainties, while the low-temperature activity remains finite over the range studied. We therefore interpret the transverse field as enabling local quantum hopping, while the dominant bottleneck is set by the noisy longitudinal-field detuning landscape.

The physical picture is illustrated in Fig. 4b. In the phenomenological spin model, the dominant microscopic process within a single activation cycle is local nearest-neighbor hopping of a domain-wall segment. Such a hop shifts the domain wall by one lattice spacing and is efficient only when the energy mismatch between the two sites involved in the process is sufficiently small. Quasi-static longitudinal-field noise modulates this mismatch from cycle to cycle^15^, so the probability of motion is controlled by the statistics of the detuning landscape rather than by the bare hopping amplitude alone.

In addition to these hopping processes, the reverse-annealing dynamics also allow single-spin flips that change the total number of particle-like spins, $\hat{\sigma }_i^z=+1$. These events correspond to particle injection in Fig. 4b and account for the gradual increase of particle density observed over many reverse-annealing cycles in Fig. 1f. They are therefore important for long-time energy relaxation and for the eventual approach toward the ordered ground state. However, on the scale of an individual activation cycle, the dominant contribution to the measured activity *R* comes from local domain-wall motion.

The corresponding material-level interpretation is shown schematically in Fig. 4c. A local polaronic rearrangement shifts the position of a CDW domain wall, while particle leakage out of the domain-wall network changes the local polaron density and enables longer-time relaxation of the metastable texture. Extended domain-wall motion can therefore arise from sequences of local hopping events, accompanied by slower particle-number relaxation. The quantum simulation supports a noise-slowed mechanism: quantum hopping provides the microscopic rearrangement channel, but quasi-static detuning suppresses this channel for most local environments and sets the probability that a domain-wall segment moves during a given activation cycle. For 1T-TaS$_2$, this provides a candidate mechanism for how intrinsically fast local hopping processes, together with slower particle leakage, can produce ultra-slow domain-wall relaxation.

## Discussion

In the broader context of quantum simulation, this work illustrates a complementary paradigm to many existing approaches. While programmable quantum platforms have successfully been used to study quantum chemistry^1^, lattice gauge theories^5^, disorder-driven localization^7^, programmable quantum matter^16,17^, and quantum phase transitions^3,4^, these studies typically explore idealized models with prospective relevance to materials. Here, instead, we use quantum simulation as a controlled environment to probe a specific dynamical mechanism motivated by a concrete solid-state system. Rather than reproducing equilibrium properties, we focus directly on non-equilibrium relaxation pathways and on the role of environmental noise in shaping microscopic dynamics.

By combining a minimal phenomenological model, effective low-energy analysis, and large-scale quantum simulation on a noisy quantum annealer, we show that discrete domain-wall rearrangements can arise from cascades of local hopping events whose intrinsic timescale is short, but whose activation is strongly suppressed by quasi-static detuning. The weak dependence of the measured reconfiguration rate on $h_x$ indicates that the relaxation dynamics are not limited by the bare hopping amplitude. Instead, the dominant bottleneck is the noisy detuning landscape that controls when local hopping channels become dynamically active. This establishes noise-slowed hopping as a mechanism capable of producing ultra-slow relaxation in systems with discrete domain-wall degrees of freedom.

For 1T-TaS$_2$, our findings suggest that the extreme separation between femtosecond electronic energy scales and hour-scale domain-wall relaxation does not require exponentially small coherent tunnelling amplitudes or coherent collective motion of large domain-wall segments. Instead, the slow dynamics may emerge naturally from intrinsically fast local hopping processes that are conditionally activated by a quasi-static detuning landscape. In this picture, domain-wall rearrangements occur when slow fluctuations of local electrostatic potentials bring neighboring CDW registries into near resonance. Once activated, local rearrangements proceed rapidly and can trigger cascades that produce the discrete domain-wall shifts observed in STM. The waiting time between such events is governed by the statistics of the detuning landscape rather than by the intrinsic polaron hopping amplitude itself. This mechanism provides a concrete, experimentally motivated candidate explanation for the weak temperature dependence and Poisson-like statistics reported at low temperatures^12^, and offers a unified framework for understanding slow dynamics without invoking coherent collective tunnelling of large domain segments.

The comparison to 1T-TaS$_2$ should therefore be understood at the level of mechanism rather than as a quantitative microscopic mapping. The phenomenological spin model does not include the full thirteenfold CDW degeneracy, explicit electronic bands, phonon modes, long-range Coulomb interactions, or the material-specific disorder landscape. Likewise, the noise spectrum of the superconducting quantum annealer is not identical to that of the material, and the reverse-annealing iteration index is not mapped onto absolute experimental time. The value of the model is instead that it isolates a generic dynamical principle: local domain-wall hopping can be intrinsically fast while the observed relaxation rate is limited by slowly varying detuning fields.

The relevance of this approach extends beyond 1T-TaS$_2$. Metastable states and their decay pathways are central to a wide range of correlated electron systems, including resistive switching oxides^18,19^, electronically phase-separated manganites^20,21^, photo-induced superconductivity^22^, and other charge-density-wave materials^23,24^. In many such systems, macroscopic functionality is governed by microscopic relaxation mechanisms that remain difficult to access experimentally. Minimal quantum simulations capable of distinguishing tunnelling-limited from noise-assisted dynamics may therefore provide valuable guidance for interpreting slow relaxation phenomena and for engineering metastable states.

An important experimental implication of our results concerns the direct detection of the quasi-static noise landscape that inhibits polaron tunnelling. Using scanning tunnelling spectroscopy (STS), it should be possible to probe the local electronic spectrum of a single site within a domain over timescales comparable to the measured reconfiguration rate. Temporal fluctuations of the local spectrum would provide direct evidence for noise-induced modulation of tunnelling barriers. In particular, correlated shifts of the local density of states on pairs of sites involved in a tunnelling event would strongly support a noise-assisted mechanism: when the local densities of states align, a tunnelling event should occur. Conversely, persistent spectral mismatches between candidate tunnelling sites would suppress reconfiguration. To be detectable, such spectral shifts must exceed the intrinsic polaron bandwidth of approximately $80\,\textrm{meV}$ in 1T-TaS$_2$^25^. Time-resolved STS measurements thus offer a concrete route to directly observe the noise processes inferred from our analysis.

Looking ahead, extending the minimal model to include fermionic degrees of freedom for electrons, longer-range interactions, explicit disorder, or coupling to phonon degrees of freedom would allow systematic investigation of how different environmental factors modify noise-assisted dynamics. On the experimental side, higher temporal resolution and controlled perturbations could further clarify the internal structure of domain-wall rearrangement cascades. Together, these directions point toward a general framework in which programmable quantum simulators serve as tools for isolating and testing microscopic dynamical mechanisms in strongly correlated quantum materials.

## Methods

### Schrieffer-Wolff transformation

The Hamiltonian after the SW transformation is $H^{'}=e^SHe^{-S}=H_0+\frac{1}{2}[S,V]+O(V^3)$, where *S* is found through solving $V+[S,H_0]=0$. We limit ourselves to a two-spin subspace that exists within an already existing domain wall, shaped as the most typical observed domain walls found in 1T-TaS$_2$ and quantum simulation. The four possible configurations within this subspace are shown in Fig. 2a. Within this subspace, we write

$$H_0 = \sum _{\psi ,\psi '} \langle \psi | H_0 | \psi ' \rangle \, |\psi \rangle \langle \psi '|, \quad \text {where }$$

$$|\psi \rangle \in \{|{-1}{-1}\rangle , |{-1}{1}\rangle , |{1}{-1}\rangle , |{1}{1}\rangle \}$$

$$H_0 = \bigg ({\begin{smallmatrix} -2 h_z + 2 J Z_h & 0 & 0 & 0 \\ 0 & 0 & 0 & 0 \\ 0 & 0 & 0 & 0 \\ 0 & 0 & 0 & 2 h_z - 2 J Z_p \end{smallmatrix}}\bigg )$$

$$V = \bigg ({\begin{smallmatrix} 0 & -h_x & -h_x & 0 \\ -h_x & 0 & 0 & -h_x \\ -h_x & 0 & 0 & -h_x \\ 0 & -h_x & -h_x & 0 \end{smallmatrix}}\bigg ).$$

The energy of the initial domain state in the top left of Fig. 2a is subtracted from $H_0$ as the reference energy. Using them, we find

$$\alpha = \frac{h_x}{2(h_z - J Z_h)}, \quad \beta = \frac{h_x}{2(h_z - J Z_p)}$$

$$S = \bigg ({\begin{smallmatrix} 0 & \alpha & \alpha & 0 \\ -\alpha & 0 & 0 & \beta \\ -\alpha & 0 & 0 & \beta \\ 0 & -\beta & -\beta & 0 \end{smallmatrix}}\bigg ),$$

which we then use to calculate

$$\lambda _{i,j} = \frac{1}{2} \left( \frac{1}{h_z - J Z_h} + \frac{1}{-h_z + J Z_p} \right)$$

$$H' = \lambda _{i,j} h_x^2 \begin{pmatrix} \displaystyle \frac{2(-h_z + J Z_p)}{J(Z_h - Z_p)} & 0 & 0 & -1 \\ 0 & 1 & 1 & 0 \\ 0 & 1 & 1 & 0 \\ -1 & 0 & 0 & \displaystyle \frac{2(h_z - J Z_h)}{J(Z_h - Z_p)} \end{pmatrix}$$

where $\lambda _{i,j}$ encodes the local number of neighboring hole-like ($\hat{\sigma }^z=-1$) $Z_h$ and particle-like spins ($\hat{\sigma }^z=1$) $Z_p$, and the indices *i*, *j* pertain to the specific choice of the two sites involved in the analysis. Crucially, first-order terms $\sim h_x$ corresponding to single spin flips are energetically forbidden (matrix elements equal to 0) within domain wall regions, as flipping a spin in the antiferromagnetic background changes the energy by either $-2h_z + 2JZ_h$ or $2h_z - 2JZ_h$. As a result, the leading contributions arise from second-order processes in $h_x$.

These second-order terms generate effective hopping between domain wall configurations. The resulting effective Hamiltonian within the subspace $|{-1}{1}\rangle , |{1}{-1}\rangle$ takes the form

$$\begin{aligned} \hat{H}_\textrm{eff}&= -h_x^2\sum _{\langle i,j \rangle } (\lambda _{i,j}\hat{\sigma }_i^+ \hat{\sigma }^-_j + \mathrm {h.c.}) + \mathcal {O}(h_x^3), \end{aligned}$$

where $\hat{\sigma }_i^+$ and $\hat{\sigma }_i^-$ are the raising and lowering operators for spins. Here, we emphasize the dependence of $\lambda _{i,j}$ on the choice of the local environment surrounding sites *i* and *j* in the 2-site model. If we reinterpret the states $|{-1}{1}\rangle , |{1}{-1}\rangle$ as occupation operator states $|{0}{1}\rangle , |{1}{0}\rangle$, we can rewrite $\hat{H}_{eff}$ using $b_i^\dagger$ and $b_i$ as the hardcore boson creation and annihilation operators, into the standard hopping term with $t \sim h_x^2$ as the effective hopping amplitude.

## Data Availability

Quantum simulation data obtained from the quantum annealer were deposited to Zenodo and are available at the following URL: https://doi.org/10.5281/zenodo.18089475.
